# Design, Implementation, and Study Protocol of a Kindergarten-Based Health Promotion Intervention

**DOI:** 10.1155/2017/4347675

**Published:** 2017-02-20

**Authors:** Susanne Kobel, Olivia Wartha, Tamara Wirt, Jens Dreyhaupt, Christine Lämmle, Eva-Maria Friedemann, Anne Kelso, Claire Kutzner, Lina Hermeling, Jürgen M. Steinacker

**Affiliations:** Division of Sports and Rehabilitation, Department of Internal Medicine II, Ulm University Medical Centre, Frauensteige 6, Haus 58/33, 89075 Ulm, Germany

## Abstract

Inactivity and an unhealthy diet amongst others have led to an increased prevalence of overweight and obesity even in young children. Since most health behaviours develop during childhood health promotion has to start early. The setting kindergarten has been shown as ideal for such interventions. “Join the Healthy Boat” is a kindergarten-based health promotion programme with a cluster-randomised study focussing on increased physical activity, reduced screen media use, and sugar-sweetened beverages, as well as a higher fruit and vegetable intake. Intervention and materials were developed using Bartholomew's Intervention Mapping approach considering Bandura's social-cognitive theory and Bronfenbrenner's ecological framework for human development. The programme is distributed using a train-the-trainer approach and currently implemented in 618 kindergartens. The effectiveness of this one-year intervention with an intervention and a control group will be examined in 62 kindergartens using standardised protocols, materials, and tools for outcome and process evaluation. A sample of 1021 children and their parents provided consent and participated in the intervention. Results of this study are awaited to give a better understanding of health behaviours in early childhood and to identify strategies for effective health promotion. The current paper describes development and design of the intervention and its implementation and planned evaluation.* Trial Registration*. The study is registered at the German Clinical Trials Register (DRKS), Freiburg University, Germany, ID: DRKS00010089.

## 1. Introduction

Although some countries report a plateau of childhood obesity, the prevalence of overweight and obesity has risen continuously during the last decades and plateaued at an unhealthy high level [1]. Main reasons for this development are a diet high in calories [2, 3] in combination with an inactive lifestyle [4] which is often already established at a young age. These behaviours have not only been linked to an increased body weight but also to chronic diseases such as cardiovascular diseases and Type 2 diabetes [5].

Therefore, interventions to promote a healthy lifestyle must start early. Risk factors are already present in young children [6] and most health behaviours develop at an early age [7], which are then carried forward into youth and adulthood [8].

There are many health promoting interventions for children—some more successful than others. Effective and sustainable health promotion programmes are multicomponent and include physical activity as well as nutrition components and are tailored to a specific target group but also include the whole environment around that group [9, 10]. For children, not only family but also important bases of socialisation such as kindergartens should be involved in health promoting interventions. There they can be reached together with their peers, independent of their background and for a longer period of time. Therefore, kindergartens are promising settings to intervene and for children to learn health behaviours, also because of the ability to stay in close contact with their parents [11].

Intervention planning for health promotion programmes should be scientifically sound and based on a theoretical model [12]. The complex process of an intervention development and its implementation and evaluation should always be guided by a planning model [13]. However, intervention planning of health promotion programmes is rarely targeted scientifically and only few developments of interventions and their contents are described in more detail [12, 14].

Based on experiences and results of the sister project “Join the Healthy Boat” in primary schools [15–17], the intervention was designed and implemented in kindergartens in the state of Baden-Württemberg, southwest Germany.

The aim of this paper is to address the development of the health promotion programme “Join the Healthy Boat – Kindergarten,” its implementation and planned evaluation including sample and methods. Development steps are shown in detail as well as the particular intervention contents and their implementation.

## 2. Materials and Methods

### 2.1. Programme Development and Implementation

**Figure 1:**
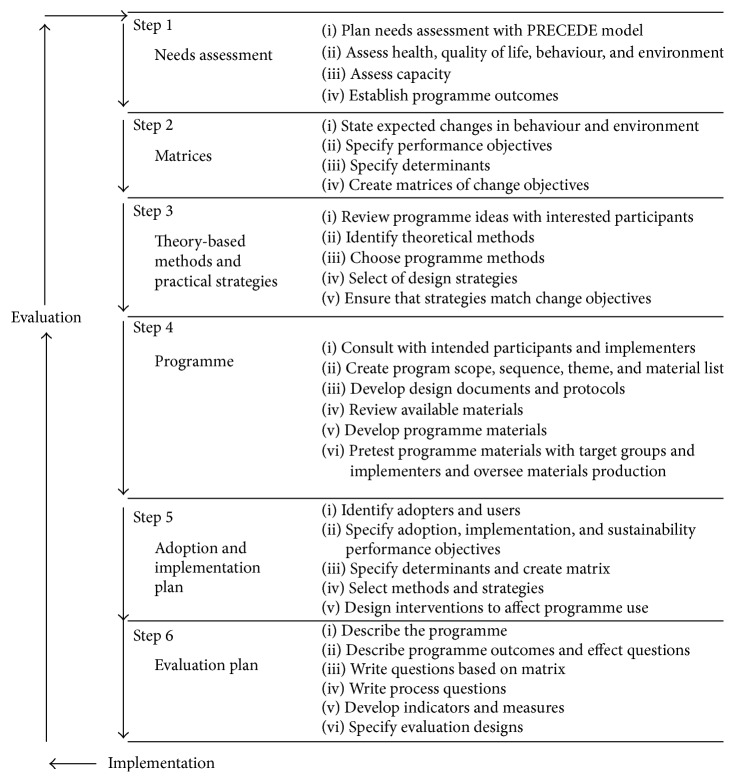
Protocol for the Intervention Mapping Approach by Bartholomew et al. [18], source: Bartholomew et al. [18].

“Join the Healthy Boat” is a multicomponent health promotion programme for kindergarten children (three to six years old) in southwest Germany, which was developed by means of Bartholomew's Intervention Mapping Approach (IMA; see Figure 1) [18].

The IMA offers guidance for a theory-based development of interventions for health promotion and includes six detailed steps: (1) needs assessment; (2) identification of intervention targets; (3) selection of theory-based intervention methods and applications; (4) integration of methods and applications into an organised programme; (5) plan for adoption, implementation, and sustainability; and (6) an evaluation plan [18].

### 2.2. Needs Assessment

During the first step “needs assessment,” literature was systematically reviewed for the topics “physical activity and leisure time activities” and “diet and nutrition” as well as for kindergarten and school-based health promotion interventions. In addition, weekly meetings of an interdisciplinary scientific team were held as well as regular focus groups with kindergarten staff and parents. For “Join the Healthy Boat,” four key topics evolved out of the needs assessment: “promotion of (everyday) physical activity,” “reduction of screen media use,” “decrease of sugar-sweetened beverages,” and “increase of fruit and vegetable intake.” These key topics were oriented towards the children's needs, specific to the target group and well implementable in a kindergarten-based health promotion programme.

### 2.3. Intervention Targets

These main topics, which were compiled during the needs assessment, were concretised and defined more thoroughly in the second step “identification of intervention targets.” With the use of so-called matrixes (extensive table templates), which are prestructured by IMA, behaviours and programme targets were developed and specified and resulted in children having to receive new knowledge and opportunities to try out new, alternative behaviours (e.g., activity games, different foods). For nutrition-specific topics, direct offers of healthy food played an important role.

### 2.4. Theory-Based Strategies

Subsequently, during step three, methods and practical strategies to attain behaviour changes were chosen, which suit target group and intervention aims (defined in step two) best. For the health promotion programme “Join the Healthy Boat,” the social-cognitive theory (SCT) by Bandura [19] and Bronfenbrenner's ecological framework for human development [20] were chosen.

The SCT assumes that behaviours are determined by personal and environmental factors as well as behaviours. Bronfenbrenner on the other hand presumes that personal relationships and social interactions influence people's (health) behaviours [20]. Therefore, measures for behaviour as well as situation/setting change can be guided. The ecological framework for human development [20] demonstrates the importance of an involvement of family and peer group as well as situational change. Newly learnt behaviours can only be maintained if they are supported by all parts of the child's living environment. The methods used in the SCT [19], such as knowledge transfer, modelling, self-evaluation, reinforcement, promotion of self-efficacy, and setting goals [21], are most suitable to achieve health-related behaviour changes in kindergarten children.

To ensure sustainability and long-term integration into the kindergarten routine, the programme applies a teacher-centred approach, which enables the implementation of profound health promoting changes in kindergarten environments and the support of children's behaviour change continuously.

### 2.5. Intervention Design and Materials

**Figure 2:**
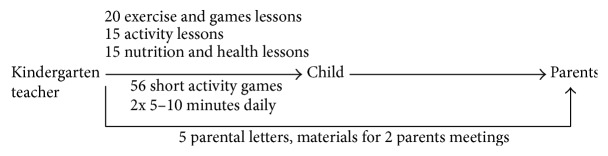
Structured overview of intervention's content.

During step four “integration of methods and applications into an organised programme,” intervention materials were designed and based on the defined programme targets and selected theoretical models from the previous steps. The materials were developed following the so-called orientation plan for kindergartens in southwest Germany [22] and in cooperation with a pedagogical advisory board consisting of kindergarten and primary school teachers as well as pedagogues. The materials are comprised of 20 exercise and games lessons as well as 30 ready-to-use ideas and action alternatives and lessons in order to get children to be more physically active and gain knowledge about their body and health as well as eat more healthily. In order to incorporate physical activity into the daily kindergarten routine, short activity games (5–7 minutes each) are part of the materials and in order to reach not only children but also their parents, materials for parents meetings and parental letters were included in three languages. During the developmental phase, intervention materials were tested for six months in 13 pilot kindergartens. An overview of the intervention's content can be found in Figure 2.

To ensure sustainability and long-term integration of “Join the Healthy Boat,” during the fifth step of IMA a train-the-trainer approach (i.e., kindergarten teachers are trained to train further kindergarten teachers) was chosen and developed [23]. For each of the 44 districts in southwest Germany, a trainer-tandem is responsible for the training courses which are held twice a year in each region. During these training courses, kindergarten teachers receive all intervention materials for free. The trainers, who are also kindergarten teachers, are trained twice a year and are intensely supported by an interdisciplinary project team at Ulm University. Apart from its cost-effectiveness [24], one advantage of this approach is that kindergarten teachers are trained by a colleague and no external expert, which enhances acceptance of the programme and enables a higher sustainability [25].

### 2.6. Study Design and Planned Evaluation

The last step of IMA guides through the planning of an evaluation of the intervention programme, which for “Join the Healthy Boat” is the so-called “Health Survey.” It was designed as a prospective, stratified, cluster-randomised longitudinal trial with an intervention and a control group and is registered at the German Clinical Trials Register (DRKS), Freiburg University, Germany, ID: DRKS00010089. The study protocol was designed in 2015 and approved by the ethics committee of Ulm University (Application Number 188/15) as well as the Ministry of Culture and Education in 2016.

Participating kindergartens were recruited from all kindergartens in southwest Germany, which have received written information about programme and study, asking interested kindergarten teachers to participate. Only kindergartens which have not previously taken part in the programme were included in the study. To ensure a similar number of children in intervention and control group, stratification of randomisation was carried out on three levels on the basis of kindergarten size, that is, kindergartens with 15 or less participating children, with 16–25 participating children, and with more than 25 participating children. Therefore, 31 kindergartens were assigned to intervention group and 31 kindergartens to control group. Children within the recruited kindergartens were eligible if they were between three and five years old at the time of baseline measurements and their parents provided a signed consent form.

### 2.7. Statistical Considerations

Further, power considerations were made prior to the study. Because of the explorative nature of this study, no adjustment for multiple testing will be made. A *p* value of less than 0.05 will be considered as significant. Subject to the number of participating kindergartens, an effect size of 0.325 (continuous outcome) can be detected with a statistical power of 0.8. The intraclass correlation coefficient was assumed as 0.05 with 40 participating children per kindergarten to reach this power. Due to the data's clustered structure, calculations will be made applying adequate statistical methods (GEE models [26]) for hierarchical data (children within kindergartens). Further details can be found in Table 1. The study's evaluation will be carried out on the basis of the intention-to-treat approach. For further assessments of effects in particular subgroups (e.g., children with migration background, overweight children) additional explorative analyses are planned. The results of all statistical tests will be interpreted in an explorative sense and not in a confirmatory way. Additionally, all main outcome variables will be adjusted for relevant socioeconomic variables (e.g., age, gender, and background).

### 2.8. Assessment Methods

An overview of all baseline analysis can be found in Table 1. Besides, intervention effects of “Join the Healthy Boat” will be assessed during the “Health Survey” on three different levels: on children's level, their parents, and kindergarten teacher/head of kindergarten.

Assessments on children's level include anthropometric measurements, such as height, body weight, waist circumference, and body fat percentage (using bioelectrical impedance analysis). Further, motor skills will be assessed using a standardised test battery including standing long jump, one-leg stand, sitting and reaching, and a 3-minute run [27]. Additionally, children's knowledge and preferences on nutrition and physical activity will be assessed in short questions as well as their well-being and interoception. On some subsamples, which parents had to provide separate consent for, physical activity will be assessed objectively over a period of six days using accelerometry and heart rate (Actiheart, CamNtech, UK), body composition will be measured using sonography, and samples of saliva and a cheek swab will be analysed for potential health risk factors and epigenetic components.

Moreover, in order to receive a more thorough picture of the child's health, parents will receive a comprehensive questionnaire rating their children's well-being, health-related quality of life and provide details on their children's and their own dietary and physical activity habits, as well as their leisure time activities. Furthermore, sociodemographic variables such as household income, parental education, and size of living space will be asked about in the questionnaire. In addition, children whose parents provided consent for saliva analyses will receive a supplementary questionnaire giving more detailed information about the child's dietary habits.

On kindergarten level, all participating kindergarten teachers as well as the head of each kindergarten will be given a questionnaire including questions about the characteristics, routines, and surroundings of the kindergarten but also about their own health behaviours and attitudes.

In order to organise and prepare those assessments, pretests were performed to plan and practice tests and procedures. Although validated questionnaires will be used, these were sent out to parents and kindergarten teachers to evaluate whether they are understandable and how long it took them to complete them. Moreover, all measurements which will be obtained from the child directly were performed at several kindergartens to ensure practicability and feasibility. Baseline measurements took place in autumn of 2016; follow-up measurements will take place one year later.

Further, as part of an extensive process evaluation, the whole course of the programme will be documented and evaluated (training courses of trainers and kindergarten teachers, implementation of intervention materials).

## 3. Results

**Figure 3:**
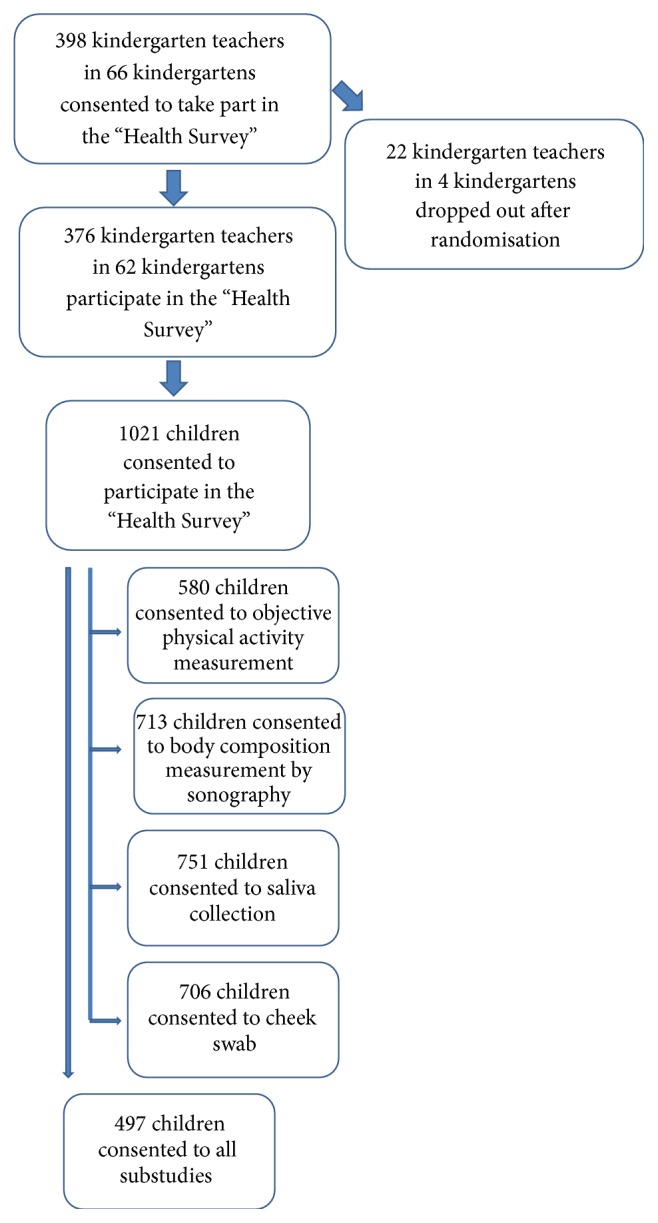
Overview of recruited children, kindergartens, and kindergarten teachers, including consent for the different substudies.

7937 kindergartens in southwest Germany, which have not previously taken part in the programme, received written information about the study and the programme. A total of 398 kindergarten teachers and their heads of 66 kindergartens provided written, informed consent for participation in the “Health Survey” (see Figure 3). After randomisation, four kindergartens with 22 kindergarten teachers dropped out, mainly due to personnel and organisation issues, resulting in 376 partaking kindergarten teachers and 62 heads of kindergartens. On average, six kindergarten teachers per kindergarten provided consent to taking part in the study, with no significant difference between intervention and control group.

Due to stratification on the basis of kindergarten size, 33 kindergartens with less than 16 participating children, 20 kindergartens with 16–25 participating children, and 9 kindergartens with more than 25 participating children are involved in the “Health Survey.”

Per kindergarten, an average of 49% of children and parents consented to participate in the study. There was no difference in participation rate in control and intervention group. In total, 1021 parents provided consent for their children's participation. Therefore, 511 children (247 boys, 264 girls, 4.1 ± 0.75 years) in 31 kindergartens were assigned to control group, whereas 510 children (277 boys, 233 girls, 4.1 ± 0.74 years) in 31 kindergartens were assigned to intervention group. There was also no significant difference between intervention and control group. Although not all children of participating kindergartens will take part in the “Health Survey,” from autumn 2016, all children in the intervention group will take part in the health promotion programme “Join the Healthy Boat,” which will be implemented by their kindergarten teachers. The control group will wait for one more year without any further intervention before starting with the programme.

In addition to the basic “Health Survey,” parents had the opportunity to agree to their children's participation in numerous substudies. Objectively assessed physical activity as well as sonography assessment of their children's body composition and the collection of saliva and a cheek swab needed the parents' specific consent. 580 parents (56.8%) want their children to take part in further objective physical activity measurements, 713 children (69.8%) have consent to have their body composition assessed using sonography, as well as 751 and 706 parents (73.6% and 69.1%) provided consent for saliva collection and cheek swab, respectively (see Figure 3). 497 parents agreed to participation in all substudies.

Baseline results of this study are expected in 2017 in order to provide a better understanding of health behaviours in early childhood and follow-up results one year later to then identify strategies for effective health promotion.

## 4. Discussion

The approach described herein of theory-based development of a health promotion intervention in kindergartens is one of few so far. In most published articles the focus lies on intervention effects but this very fundamental and important step is hardly mentioned or not at all, although particularlly development steps like these should be made available to other programme planners in order to standardise interventions and enable enhancements of such interventions for health promotion.

### 4.1. Intervention Development

For the development of the “Join the Healthy Boat” intervention, the structured planning model “intervention mapping” [18] was used. It is a helpful approach in order to receive systematised support during the planning phase and to concretise aims, methods, and the whole intervention. However, the use of IMA is very time-consuming and complex—sufficient lead time is as necessary as access to different scientific qualifications. In order to develop and implement the programme “Join the Healthy Boat,” physicians, sport scientists, psychologists, health scientists, nutritionists, and pedagogues had to work together, which are disciplines that cover all competences for health promotion of the IMA postulates. Further, the previously mentioned high expenditure of time added up to 16 months from first considerations to the actual implementation of the programme in kindergartens.

### 4.2. Setting

The setting kindergarten is very well suited to implement intervention programmes for health promotion since it is possible to realise behaviour change (e.g., knowledge transfer; increase of self-efficacy) as well as environmental change (e.g., offer of healthy beverages and nutrition; gardens/surroundings which stimulate children to be physically active). That way, children get backing and motivation from their peers in all emerging health-related changes. Moreover, for children that age (kindergarten: 3 to 6 years), the SCT is an ideal framework since its implementation strategies such as model learning can be accomplished very well in the kindergarten setting [28]. The SCT is recommended and used as theoretical framework by many successful interventions for health promotion [28]; yet, its specific use and implementation or those of other behaviour change theories in context of kindergarten or school-based health promotion interventions are very rarely described in detail [10, 12].

### 4.3. Intervention Materials and Design

The developed intervention is extensive and contains differentiated materials which are suitable for children of the targeted age and can be implemented directly in kindergarten's daily routine. The detailed, elaborated intervention supports kindergarten staff during their regular activity since the key aspects of the intervention (physical activity and nutrition and leisure time activities) are topics reported in the orientation plan for kindergartens in southwest Germany [22]. The intervention materials integrate physical activity directly into the kindergarten routine since studies show that successful interventions for health promotion depend on possibilities of implementing activity in situ [29]. Ideally, the included activity exercises are used straight after the daily morning circle and become part of a ritualisation and rhythmisation, which are important in kindergarten. All materials support already existing elements of the daily kindergarten routine such as regular activity sessions, trips, and joint cooking and baking activities.

In order to reach all families, even those with migration background or families from an underprivileged educational background, materials for parental work were designed appealingly and drafted in easy and plain language. These also offer specific action alternatives as well as health-related information. The first parental letter is actually a letter to the children, which children receive and should be read to them by their parents. This is an attempt to get families to really concern themselves with those parental letters. Furthermore, materials and training sessions provide ideas and opportunities (posters designed by children, activities such as a joint healthy breakfast, parental letters, etc.) to present health relevant information to parents without them having to feel imposed upon. All parental letters are also translated into Turkish and Russian.

Even though there are some programmes addressing physical activity and nutrition in kindergartens, the needs assessment demonstrated that long-term interventions without external staff are very rare but desired. The close peer-to-peer train-the-trainer approach of the programme can be seen as an innovation. Kindergarten teachers are coached by a trainer with expert knowledge of elementary pedagogy and physical activity and nutrition in order to enable them to implement the intervention autonomously and on the long-term. Key focus of the training sessions and materials is always the presentation and introduction of easily implemented action alternatives. In order to facilitate that, the intervention materials are anchored in a pirate story using puppets, short stories, illustrations, and so forth which are designed appealingly and suitably for children.

Besides, literature advises a duration of at least one year for successful kindergarten-based interventions for health promotion [10, 30]. The intervention “Join the Healthy Boat” was designed to reach children for one year or longer, ideally during their whole time in kindergarten. Since the programme does not need any external staff, trained kindergarten teachers can continue the programme without any further costs.

### 4.4. Evaluation and Its Challenges

However, the upcoming programme's evaluation (“Health Survey”) is scheduled for one year, with the possibility of tracking participating children through their childhood and adolescence. Its recruitment process was challenging and hindered by numerous aspects but mostly because interested kindergarten teachers had to volunteer to take in a rather extensive programme with an equally comprehensive evaluation. Most kindergartens in Germany already take part in some programmes—whether it is health promotion, language support, or dental prevention—which are all time-consuming. Further, the constant shortage of staff makes matters more difficult and adds to a reluctance to agree to any further work. Additionally, timing might not have been ideal since the new orientation plan for kindergartens, which the teachers need to adhere to, adds further work load and requires plenty of restructuring within the kindergarten setting. However, although the programme started two years ago and 1209 kindergarten teachers have already been trained and implemented “Join the Healthy Boat” in their kindergartens, it was still possible to recruit 62 kindergartens with 1021 participating children and 376 partaking kindergarten teachers, who are all willing to implement the programme in full and also document and evaluate certain aspects of the intervention during that year.

Meanwhile, the “Health Survey” will—hopefully—provide detailed information on determinants and influencing health behaviours of kindergarten children and their parents and kindergarten teachers as well as relevant data on the programme's effectiveness and acceptance, which may support decisions for public health policies regarding further health promotion programmes on kindergarten level.

### 4.5. Strengths and Limitations

Although the “Join the Healthy Boat” intervention has several strengths, its limitations also have to be considered. The standardisation of its intervention materials and teacher training sessions as well as evaluation and implementation protocols is a major strength of this study. Nevertheless, irrespective of several objective assessment methods which will be used in the “Health Survey,” most data will be based on self-report by either kindergarten teachers or parents, which may be prone to social desirability or recall bias, especially considering that teachers and parents knew about the aims of this study. Moreover, since only very few of the addressed kindergartens were prepared to take part in the study, those then might be more determined or committed kindergarten teachers and representative results cannot be expected. However, since kindergartens and their teachers and children did not differ in control and intervention group, intervention effects should not be affected. Likewise, even though the recruited sample size is relatively large and spread all over the southwest of Germany, generalisation of results will not be possible. Also, a one-year intervention might be not long enough and the one follow-up assessment the following year might not be sufficient to provide enough details and insight on the impact of the programme. Still, due to the application of IMA it was possible to document the development process of the programme in detail and, therefore, that information can now be made available to other researchers wanting to develop interventions like this. Because of the transparency, a standardisation and comparability are possible, which increases an evidence-based public health requests [31].

### 4.6. Considerations to Be Made When Planning a Setting-Based Intervention

When developing and implementing such a multicomponent health promotion programme, some things should be considered beforehand. As mentioned previously, the use of IMA is desirable as very structured but also very time-consuming and complex; therefore, because lead time is necessary when using that framework in order to develop a health promotion programme, also, access to different scientific qualifications is advantageous if not essential, if different components want to be included in such an intervention.

Further, numerous and intense talks in focus groups with potentially participating people are crucial, since programmes which are not tailored directly towards the needs of the targeted group will not be accepted and implemented by them.

Moreover, in order to plan and recruit for an appropriate evaluation, additional considerations have to be made. First and foremost, an open and honest communication with participants (in this case: parents and kindergarten teachers) about what they have to expect is essential. Kindergarten teachers have to know exactly how much time it will take them to prepare and implement the materials and how much extra time the evaluation will take. Furthermore, the design of attractive and appealing promotion and recruitment materials, which contain all aspects of the evaluation in easy language, has shown to be important in order to reach all possible participants. Also, plenty of time is necessary in order to recruit sufficient kindergartens and to explain to all participants the necessity of a control group. Besides, the choice of measurements and methods is a complex one which should not be underestimated.

## 5. Conclusion

In summary, “Join the Healthy Boat” was developed by a multidisciplinary team using the intervention mapping approach [18] utilising Bandura's social-cognitive theory [19] and the ecological framework for human development by Bronfenbrenner [20]. During the first two years, 618 kindergartens and 1209 kindergarten teachers were trained and implemented the programme into the children's daily routines.

For the upcoming evaluation of the here described intervention, 62 kindergartens, 376 kindergarten teachers, and 1021 children could be recruited. The outcome and process evaluation along with other variables for the “Join the Healthy Boat” intervention will be assessed shortly and its results are awaited to give a better understanding of health behaviours in early childhood and to identify strategies for effective health promotion.

## Figures and Tables

**Table 1a:** (a) Main outcomes

Outcome	Statistical analysis
Change in variables for nutrition (consumption of sugar-sweetened beverages, fruit, vegetables, high-calorie food; all variables are ordinal)	GEE model for follow-up measurement

Change in child's time spent with screen media (hours per week, ordinal/quasi-continuous variables)	GEE model for follow-up measurement

Change in child's physical activity/energy expenditure (physical activity: dichotomous, energy expenditure: continuous variables)	GEE model for follow-up measurement

Change in health knowledge and attitude of parents and kindergarten teachers (nominal/ordinal variables)	GEE model for follow-up measurement

**Table 1b:** (b) Secondary outcomes

Outcome	Statistical analysis
Change in anthropometric parameters (waist circumference, waist-to-height ratio, BMI, subcutaneous fat; all variables are continuous)	GEE model for difference between follow-up and baseline

Change in child's quality of life (EQ5D-Y Proxy Version, KINDL parent's version; continuous variables)	GEE model for difference between follow-up and baseline

Child: change in days sick leave, doctor's consultation, hospitalisation; parents: absence from work due to children's illness (continuous variables)	GEE model for difference between follow-up and baseline

Change in child's motor skills (continuous variables)	GEE model for difference between follow-up and baseline

Environment of kindergarten and change in environment of kindergarten (continuous and categorical variables; measured on kindergarten level)	*t*-test or Wilcoxon test as appropriate, respectively, *χ*^2^-test or Fisher's exact test as appropriate for baseline and difference between follow-up and baseline

Change in laboratory parameters (saliva, cheek swab): metabolic and inflammatory parameters, for example, IL-6, TNF*α*, leptin, epigenetic investigation (continuous variables)	GEE model for difference between follow-up and baseline
